# COVID-19 vaccine inequality: A global perspective

**DOI:** 10.7189/jogh.12.03072

**Published:** 2022-10-14

**Authors:** Moosa Tatar, Jalal Montazeri Shoorekchali, Mohammad Reza Faraji, Mohammad Abdi Seyyedkolaee, José A Pagán, Fernando A Wilson

**Affiliations:** 1 Center for Value-Based Care Research, Cleveland Clinic, Cleveland, Ohio, USA; 2 Institute for Humanities and Cultural Studies, Tehran, Iran; 3 Department of Computer Science and Information Technology, Institute for Advanced Studies in Basic Sciences (IASBS), Zanjan, Iran; 4 Department of Economics, Faculty of Economics and Administrative Sciences, University of Mazandaran, Babolsar, Iran; 5 Department of Public Health Policy and Management, School of Global Public Health, New York University, New York, New York, USA; 6 Matheson Center for Health Care Studies, University of Utah, Salt Lake City, Utah, USA; 7 Department of Population Health Sciences, University of Utah, Salt Lake City, Utah, USA; 8 Department of Economics, University of Utah, Salt Lake City, Utah, USA

By the end of 2021, more than 12 billion COVID-19 vaccine doses have been globally distributed and administered [1]. However, nearly one million new daily cases and more than two thousand new daily deaths were reported by July 2022. The best way to slow the spread of the COVID-19 virus and the most effective way to prevent severe illness, hospitalizations, and death is to get vaccinated [2]. From the beginning of the global COVID-19 vaccination campaign, the World Health Organization (WHO) and the COVID-19 Vaccines Global Access (COVAX) initiative strived to guarantee fair and equitable vaccine rollouts worldwide. The WHO aims to achieve global access to the COVID-19 vaccines by mid-2022 with the goal of vaccinating 40% of the population of every country by the end of 2022 [3]. Nevertheless, substantial unequal COVID-19 vaccine distribution was reported a few months after the first public COVID-19 vaccination (March 31, 2021), and the emergence of new SARS-COV-2 variants has highlighted this issue [4]. We used Gini coefficients to measure the degree of COVID-19 vaccine inequality throughout the globe.

We created a data set with 222 countries and territories, accounting for approximately 99.7% of the world population in six continents: Africa (55), Asia (50), Europe (50), North America (34), Oceania (20), and South American (13). We obtained COVID-19 vaccination dose data up to December 7, 2021, and population data from the Our World in Data website [5]. We included COVID-19 vaccination data from December 8, 2020, to June 7, 2021 (six months after the first public COVID-19 vaccination) and conducted our analysis on vaccine inequality at three levels: 1) at the global level with 222 countries and territories, 2) at the continent level, between the six continents, and 3) within continents, for each continent separately. Additionally, we repeated all the analyses using COVID-19 vaccination data to December 7, 2021 (one year after the first public COVID-19 vaccination).

The Gini coefficient is a commonly utilized inequality index for measuring the degree of distribution inequality. It summarizes the distribution of a targeted variable (ie, doses of COVID-19 vaccines) into a single statistic by comparing cumulative proportions of the population against cumulative proportions of vaccines they receive and indicating a relationship between the proportion of a population and the proportion of vaccines distributed. If the proportions of population and vaccines are equal (ie, each country/continent receives an equal share of vaccines based on their population size), then equality exists. If the proportions of population and vaccines are not equal, then inequality exists.

The Gini Coefficient ranges from “0” to “1”, in which “0” represents the perfect equal distribution, and “1” represents perfect unequal distribution [6]. Therefore, a greater Gini coefficient means a greater degree of inequality. There is no defined standard inequality cutoff; however, it is recognized that a Gini coefficient of less than 0.2 is considered perfect equality, a Gini coefficient of 0.2 to 0.4 as relative inequality, and 0.4 to 0.5 as high inequality. A Gini coefficient above 0.5 is regarded as severe inequality. The analyses were conducted in 2022 using the Stata SE. V.15.1 (College Station, Texas) statistical package.

**Table 1** reports Gini coefficients for COVID-19 vaccinations. World Gini coefficients for COVID-19 vaccines were 0.91 and 0.88 on June 7 and December 7, 2021, respectively, denoting severe COVID-19 vaccine inequality. The Gini coefficients between continents on June 7 and December 7, 2021, were 0.57 and 0.61, respectively, and indicate severe inequality between continents. Results show North America has the highest Gini coefficients (0.91 on December 7, 2021) and South America has the lowest Gini coefficients (0.61 on December 7, 2021).

**Table 1:** COVID-19 vaccination inequality

Geography	Share of the world population	Total vaccination % until June 7, 2021	Total vaccination % until December 7, 2021	Gini coefficient until June 7, 2021	Gini coefficient until December 7, 2021
Africa	17.47	1.63	3.08	0.79	0.73
Asia	59.26	56.13	67.80	0.91	0.86
Europe	9.56	17.97	11.92	0.74	0.73
North America	7.55	17.94	9.26	0.94	0.91
Oceania	0.55	0.29	0.61	0.88	0.88
South America	5.62	6.04	7.31	0.74	0.68
Continents	100	100	100	0.57	0.61
World*	100	100	100	0.91	0.88

*Including 222 countries and territories covering approximately 99.7% of the world's population in six continents: Africa (55), Asia (50), Europe (50), North America (34), Oceania (20), and South American (13). Gini coefficient ranges from “0” to “1”, in which “0” represents the perfect equal distribution and “1” represents perfect unequal distribution.

Our results are consistent with prior research that reported extreme disparities and inequalities during the COVID-19 pandemic (eg, in testing, infections, hospitalizations, and mortality) [7,8]. A recent study using data up to March 31, 2021 (approximately four months after the first public COVID-19 vaccination on December 6, 2020) reported a global Gini coefficient of 0.88 for COVID-19 vaccinations [4]. Our results show that not only has the distribution of COVID-19 vaccinations not improved, but the inequality of COVID-19 vaccinations was also more severe by December 7, 2021. Moreover, while the distribution of vaccines was slightly less severe between continents (0.61), the inequality within continents was very severe. However, the inequality is somewhat less severe within Europe (0.73). Additionally, Asia has 59.3% of the world's population, and, even though 67.8% of the world’s vaccines were distributed in Asia, this distribution was very unequal across Asian countries (Gini coefficient was 0.86 on December 7, 2021). The inequality in vaccines was similarly high in Oceania (0.88). On the other hand, with 17.5% of the world's population and administering only 3.1% of the world’s supply of vaccines, the distribution of vaccines in Africa was less severely unequal compared to Asia (Gini coefficient was 0.73 on December 7, 2021). Europe and South America received relatively proportionate shares of COVID-19 vaccines compared to their populations, and their distributions had somewhat less inequality than Asia, Oceania, or North America.

While the United Nations Children's Fund (UNICEF), on behalf of the COVAX initiative, delivered nearly one billion COVID-19 vaccines to low- and middle-income countries in 2021 [9], our analysis suggests that the global vaccine distribution has been severely unequal. With the threat of recurring waves of potential “escape variants” of SARS-COV-2, this continued inequitable access to vaccines creates a long-term threat to global population health. This implies that the COVAX initiative efforts have not been sufficient to address the COVID-19 vaccine inequality globally, and renewed coordinated efforts are needed to boost vaccine rollouts in low-and middle-income countries [10].

**Figure:**
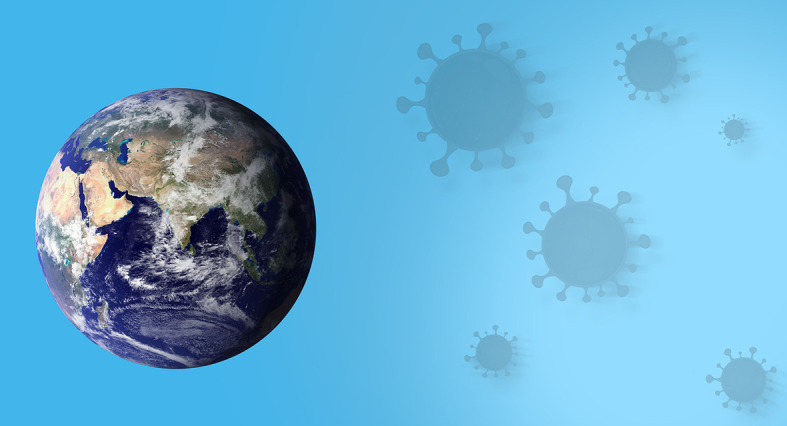
Photo: COVID-19 pandemic. Source: Free to use under Pixabay license. Available at: https://pixabay.com/illustrations/earth-virus-attack-concept-idea-5681017/.

High-income countries should expand their COVID-19 response strategies to the global level. These countries should adjust their national vaccine distribution policies and allocate a certain portion of their vaccine supplies based on ongoing morbidity or mortality rates worldwide. This approach not only would reduce vaccine disparities in low-and middle-income countries, especially in Africa, but also can delay or eliminate new COVID-19 waves worldwide and decrease the total number of infected individuals globally.

High-income countries have been accused of stockpiling COVID-19 vaccines for booster shots; however, the World Health Organization recommends boosters only for individuals with health issues and older individuals. In fact, allocating COVID-19 vaccines to low-and middle-income countries is considered a charity/donation, and high-income countries refuse to support sharing of their vaccine technology. However, if high-income countries realize COVID-19 vaccines are a global public good, and that allocating COVID-19 vaccines to countries that cannot purchase vaccines in adequate quantities will eventually benefit them via spillovers and positive global externalities, this may increase their willingness to donate/allocate COVID-19 vaccines to low-and middle-income countries. The potential positive global externality could be lower probabilities of emerging potential escape COVID-19 variants and decreasing the number of new cases worldwide.
